# Gene expression of *Paracoccidioides* virulence factors after interaction with macrophages and fibroblasts

**DOI:** 10.1590/0074-02760200592

**Published:** 2021-03-26

**Authors:** Jaqueline Derissi Braz, Janaina de Cássia Orlandi Sardi, Nayla de Souza Pitangui, Aline Raquel Voltan, Ana Marisa Fusco Almeida, Maria José Soares Mendes-Giannini

**Affiliations:** 1 Universidade Estadual Paulista, Faculdade de Ciências Farmacêuticas, Departamento de Análises Clínicas, Laboratório de Micologia Clínica, Araraquara, SP, Brasil; 2 Universidade Federal de Mato Grosso do Sul, Faculdade de Ciências Farmacêuticas, Alimentos e Nutrição, Campo Grande, MS, Brasil; 3 Universidade de São Paulo, Faculdade de Medicina de Ribeirão Preto, Departamento de Biologia Celular e Molecular, Ribeirão Preto, SP, Brasil

**Keywords:** *Paracoccidioides* spp., adhesin genes, virulence factors, M form, Y form

## Abstract

**BACKGROUND:**

Paracoccidioidomycosis (PCM) is a systemic mycosis with high prevalence in Latin America that is caused by thermodimorphic fungal species of the *Paracoccidioides* genus.

**OBJECTIVES:**

In this study, we used quantitative polymerase chain reaction (qPCR) to investigate the expression of genes related to the virulence of *Paracoccidioides brasiliensis* (Pb18) and *P. lutzii* (Pb01) strains in their mycelial (M) and yeast (Y) forms after contact with alveolar macrophages (AMJ2-C11 cell line) and fibroblasts (MRC-5 cell line).

**METHODS:**

The selected genes were those coding for 43 kDa glycoprotein (gp43), enolase, glyceraldehyde-3-phosphate dehydrogenase (GAPDH), 14-3-3 protein (30 kDa), phospholipase, and aspartyl protease.

**FINDINGS:**

In the Pb18 M form, the aspartyl protease gene showed the highest expression among all genes tested, both before and after infection of host cells. In the Pb18 Y form after macrophage infection, the 14-3-3 gene showed the highest expression among all genes tested, followed by the phospholipase and gp43 genes, and their expression was 50-fold, 10-fold, and 6-fold higher, respectively, than that in the M form. After fibroblast infection with the Pb18 Y form, the 14-3-3 gene showed the highest expression, followed by the phospholipase and aspartyl protease genes, and their expression was 25-fold, 10-fold, and 10-fold higher, respectively, than that in the M form. Enolase and aspartyl protease genes were expressed upon infection of both cell lines. After macrophage infection with the Pb01 Y form, the 14-3-3 gene showed the highest expression, followed by the phospholipase and aspartyl protease genes, and their expression was 18-fold, 12.5-fold, and 6-fold higher, respectively, than that in the M form.

**MAIN CONCLUSIONS:**

In conclusion, the data show that the expression of the genes analysed may be upregulated upon fungus-host interaction. Therefore, these genes may be involved in the pathogenesis of paracoccidioidomycosis.

Paracoccidioidomycosis (PCM) is a systemic granulomatous disease caused by a complex group of thermally dimorphic fungi within the genus *Paracoccidioides*. This disease is endemic in Latin America, and although it is not classified as a neglected tropical disease (NTD) by the World Health Organization (WHO), it meets all NTD criteria.^1^ Currently, at least 10 million people are infected.^2^ ^,^ ^3^ ^,^ ^4^ PCM may affect any organ in the body, predominantly the lungs, which are organs rich in cells of the mononuclear phagocyte system.^5^ ^,^ ^6^ ^,^ ^7^ Even after successful antifungal treatment, most patients with a chronic form have sequelae, including pulmonary fibrosis. Therefore, these patients have respiratory problems that often hinder them from exercising their professions and activities.^8^ ^,^ ^9^

Geographical evidence and genetic variability suggest that *Paracoccidioides* spp. could be composed of several genetic groups.^4^ The disease may occur in areas that extend from Mexico to Argentina, and the species currently recognised are *Paracoccidioides brasiliensis*, *P. americana*, *P. restrepiensis*, *P. venezuelensis*, and *P. lutzii*.^10^

*P. brasiliensis*, the most studied species, has been considered a facultative intracellular organism that can interact with epithelial cells and macrophages.^11^ Macrophages constitute one of the primary defense mechanisms against infection by this fungus.^2^ ^,^ ^12^ ^,^ ^13^ Phagocytes recognise the fungal cell wall, which is comprised of pathogen-associated molecular patterns (PAMPs) that are not synthesised by the host.^14^ ^,^ ^15^ The surface molecules of the organism are involved in strategies to evade the host’s immune system and ensure survival. This is closely associated with transcriptional control involving several regulatory pathways.^14^

Various components of *Paracoccidioides* spp., such as adhesins and others, may play a relevant role in pathogenesis.^16^ ^,^ ^17^ ^,^ ^18^ ^,^ ^19^ ^,^ ^20^ ^,^ ^21^ de Oliveira et al.^22^ evaluated the gene expression of different adhesins, including 14-3-3 protein, GP43, enolase (ENO), glyceraldehyde 3-phosphate dehydrogenase (GAPDH), triosephosphate isomerase (TPI), and malate synthase (MLS), after mice infection with *P. brasiliensis* and *P. lutzii*.^22^ The study found differences among the two species, and that 14-3-3 and ENO showed the highest expression*.* Other virulence factors include proteinases and phospholipases.^23^ Transcriptome analysis of *P. brasiliensis* yeast cells (Y form) derived from infected mice^24^ ^,^ ^25^ and after incubation in human blood and plasma^26^ ^,^ ^27^ revealed a positively regulated serine protease transcript.

This serine proteinase (gi734682187), previously characterised during fungal growth upon nitrogen deprivation,^25^ was accumulated in Y cells after lung infection, probably enabling the pathogen to enter lung tissue and spread.^28^ ^,^ ^29^ ^,^ ^30^ ^,^ ^31^ *P. brasiliensis* interaction with pneumocytes and keratinocytes *in vitro* may be correlated with pulmonary fungal entry events and dissemination.^32^ ^,^ ^33^ ^,^ ^34^ Thus, the study of the interaction of *Paracoccidioides* spp. with lung cells and macrophages in vitro may constitute a fundamental approach for understanding the initial stages of this fungal infection.

The majority of the studies on fungal-host interactions have been performed on the Y form of the fungus.^21^ ^,^ ^35^ ^,^ ^36^ ^,^ ^37^ However, to study the early stage of the disease and fungal adaptation to the host’s conditions, it is best to comparatively study the M and Y forms. Thus, this study aimed to compare virulence gene expression between the M and Y forms of *P. brasiliensis* (Pb18) and *P. lutzii* (Pb01), using quantitative polymerase chain reaction (qPCR) before and after infection of alveolar macrophages and human lung fibroblasts.

## MATERIALS AND METHODS

*Isolates* - *P. brasiliensis* 18 (ATCC 32069) and *P. lutzii* 01 (ATCC MYA-826) from the collection of the Laboratory of Clinical Mycology, Faculty of Pharmaceutical Sciences of UNESP, Araraquara (Brazil) were used in the M and Y forms. Both species were grown in Fava-Netto’s semi-solid medium at 23ºC (M form) and 37ºC (Y form).

*Cell culture* - AMJ2-C11 (mouse alveolar macrophages) and MRC-5 (human lung fibroblasts) cell lines were obtained from the Cell Bank of Rio de Janeiro and cultured in HAM-F12 medium at 37ºC and 5% CO_2_.

*Culture of macrophages and lung fibroblasts* - Macrophages and lung fibroblasts were grown in plastic bottles containing Dulbecco’s Modified Eagle Medium (DMEM, Sigma-Aldrich, Brazil) supplemented with foetal bovine serum (FBS), 100 µg/mL streptomycin, and 100 U/mL penicillin (Gibco BRL, Life Technologies, Rockville, MD, USA), and maintained at 36.5ºC. After three to four days, the bottles were subjected to trypsinisation. For this, the monolayer was first washed with 1.0 mL sterile phosphate-buffered saline (PBS, 0.05 M, pH 7.2), followed by addition of 1 mL ATV solution (0.2% trypsin and 0.02% EDTA - Sigma^®^). After 1-2 min, the cells were homogenised with varying amounts of the corresponding medium supplemented with FBS. In this step, ATV was neutralised by the FBS present in the culture medium. The obtained cell suspension was transferred to other cylinders, resulting in a cell concentration of 10^6^ cells/mL.

*Preparation and standardisation of the inocula* - *P. brasiliensis* isolate 18 (ATCC 32069) and *P. lutzii* strain 01 (ATCC-MYA-826) were maintained on the solid culture medium Fava-Netto (Fava Netto) at 36*º*C for the yeast form and at 23*º*C for the mycelium form during five and 15 days, respectively. The cell viability was assessed by using trypan blue in a hemocytometer chamber. The preparation of the inoculum was the same as used in the M and Y form. The Paracoccidioides spp. growth was washed three times with PBS buffer after the mycelium was fragmented by glass beads using a vortex and filtered through glass wool. Besides, the cell clumps were separated by repeated passages through an 18-gauge needle (Becton Dickinson, Brazil) into a 5 mL syringe (Becton Dickinson, Brazil). The suspension was filtered through 40 µm nylon cell strainers (Corning, USA) to obtain cells of homogeneous sizes. Enough sterile PBS and fungus were employed to obtain a homogeneous turbid suspension, corresponding to 1 x 105 cells/mL, according to the MacFarland scale and adjusted by spectrophotometric reading [optical density (OD) = 0.5].

*Infection of human lung fibroblasts and mouse alveolar macrophages by P. brasiliensis and P. lutzii* - Y and M form of both species was employed for infection assays. After the formation of the cell monolayer, the culture medium was discarded, and 3 ml of inoculum suspension was added. The bottles containing infected lung cells were incubated with the suspension for 5 h, and bottles containing macrophages were incubated for 6 hours. A total of 1 x 10^5^ cells/mL (Y or M form) were added to the cells to obtain a yeast/macrophage or yeast/fibroblast ratio of 1:1. After the infection period, the cells were removed with a scraper (scraper) and transferred to a Falcon^®^. The cells were washed 3x with PBS and stored at - 80ºC for later RNA extraction.

Differential gene expression analysis by quantitative PCR

*Extraction of total RNA* - In independent experiments, fungal cells were isolated and macerated after freezing with liquid nitrogen in a Falcon tube containing 3 mL Trizol and glass beads. The sample was shaken vigorously by vortexing for 15 min and, after 10 min at rest, centrifuged at 10,000 g for 10 min. The supernatant was transferred to a new tube and 200 µL chloroform was added for each 750 µL of the recovered Trizol. The tube was shaken vigorously, left to stand for 10 min, and centrifuged at 10,000 g for 15 min. Then, the supernatant was transferred to a new tube and 250 µL isopropanol, 250 µL of a 0.4 M sodium citrate solution, and 0.8 M sodium chloride were added to 750 µL Trizol. The samples were stirred gently and allowed to stand for 10 min at room temperature. Immediately after centrifugation at 10,000 g for 30 min, the supernatant was discarded, and the pellet was washed with 75% ethanol and centrifuged at 10,000 g for 5 min. The supernatant was again discarded, and the precipitate was dried in SpeedVac equipment. After drying, the pellet was re-suspended in 40 µL of a 0.01% diethyl pyrocarbonate (DEPC) solution in H_2_O, and the samples were stored at -80ºC.^38^ ^,^ ^39^

*Synthesis of cDNA* - The synthesis of the first cDNA tape was made in a reaction containing 1 µg total RNA and 10 pmol/μL primer (5’-AAGCAGTGGTATCAACGCAGAGTACGCGGG-3’), and the mixture was heated for 2 min at 72ºC. Then, 2 μL of a mixture of dNTPs (10 mM), 2 μL of RNaseOUT (40 U/μL) (Invitrogen, Carlsbad, California, EUA), 0.4 µL DTT (100 mM), 1.2 µL magnesium chloride (50 mM), 2 μL reverse transcriptase (200 U/μL) (Superscript II RT - Invitrogen), and 4 μL buffer 5X (250 mM Tris-HCl, 375 mM KCl, 15 mM MgCl_2,_ pH 8.3) were added. This reaction was incubated at 42ºC for 1 h and 30 min. Thereafter, 80 μL TE (10 mM Tris-HCl, 1 mM EDTA, pH 8.0) was added and the mixture was heated for 10 min at 72ºC.^40^

*Quantitative PCR* - Differential gene expression between the Y and M forms of both species after macrophage and fibroblast infection was examined using qPCR with specific primers designed using Primer 3 software (Table).

**TABLE:** Specific primers used for the quantitative polymerase chain reaction (qPCR) assay

	Primers
gp43	Sense 5´-CTTGTCTGGGCCAAAAACTC-3´
	Antisense 5´-GCCAGGGTTTGTTTGACTGT-3´
Enolase	Sense 5´-TAGGCACCCTCACTGAATCC-3´
	Antisense 5´-GCTCTCAATCCCACAACGAT-3´
Phospholipases	Sense 5´-TGTTGGTGCGATCAAAAGAC-3
	Antisense 5´-GGATACGACGTCGCCACTAT-3
Aspartyl protease	Sense 5´-AAAGGAAACACGGAAACACG-3
	Antisense 5´-CGTTCCTGAGACGGTGGTAT-3
14-3-3 (30 kDa)	Sense 5´GTTCGCTCTTGGAGACAAGC-3´
	Antisense 5´AGCAACCTCAGTTGCGTTCT-3´
GAPDH	Sense 5´-AAATGCTGTTGAGCACGATG-3´
	Antisense 5´-CTGTGCTGGATATCGCCTTT-3´

The reactions were performed using 1 µL cDNA, 10 µL of maximum SYBR Green/ROX qPCR Master Mix (2X) (Thermo Scientific, Waltham, Massachusetts, EUA), and 0.5 mM of each primer, to which water was added until the reaction volume was 20 µL. The reactions were performed at an initial temperature of 50ºC for 2 min, followed by 10 min at 95ºC and 40 cycles at 95ºC for 15 s, and annealing and synthesis at 60ºC for one min. PCR analysis of the melting curve was performed to confirm the presence of a single specific product. The reactions were performed in triplicate with an Applied Biosystems 7500 cycler machine. Variations in mRNA expression were calculated using the 2-ΔCT formula, where ΔCT is the difference between the targets and the reference gene β-tubulin, following previous studies performed by several authors.

*Statistical analysis* - All data were subjected to the Shapiro-Wilk test to check for normal distribution. Differences in gene expression between the M and Y forms of Pb18 and Pb01 after macrophage and lung fibroblast infection was assessed using one-way analysis of variance (ANOVA) with Tukey’s multiple comparison tests and a significance level of 5%.

## RESULTS

**Fig. 1:**
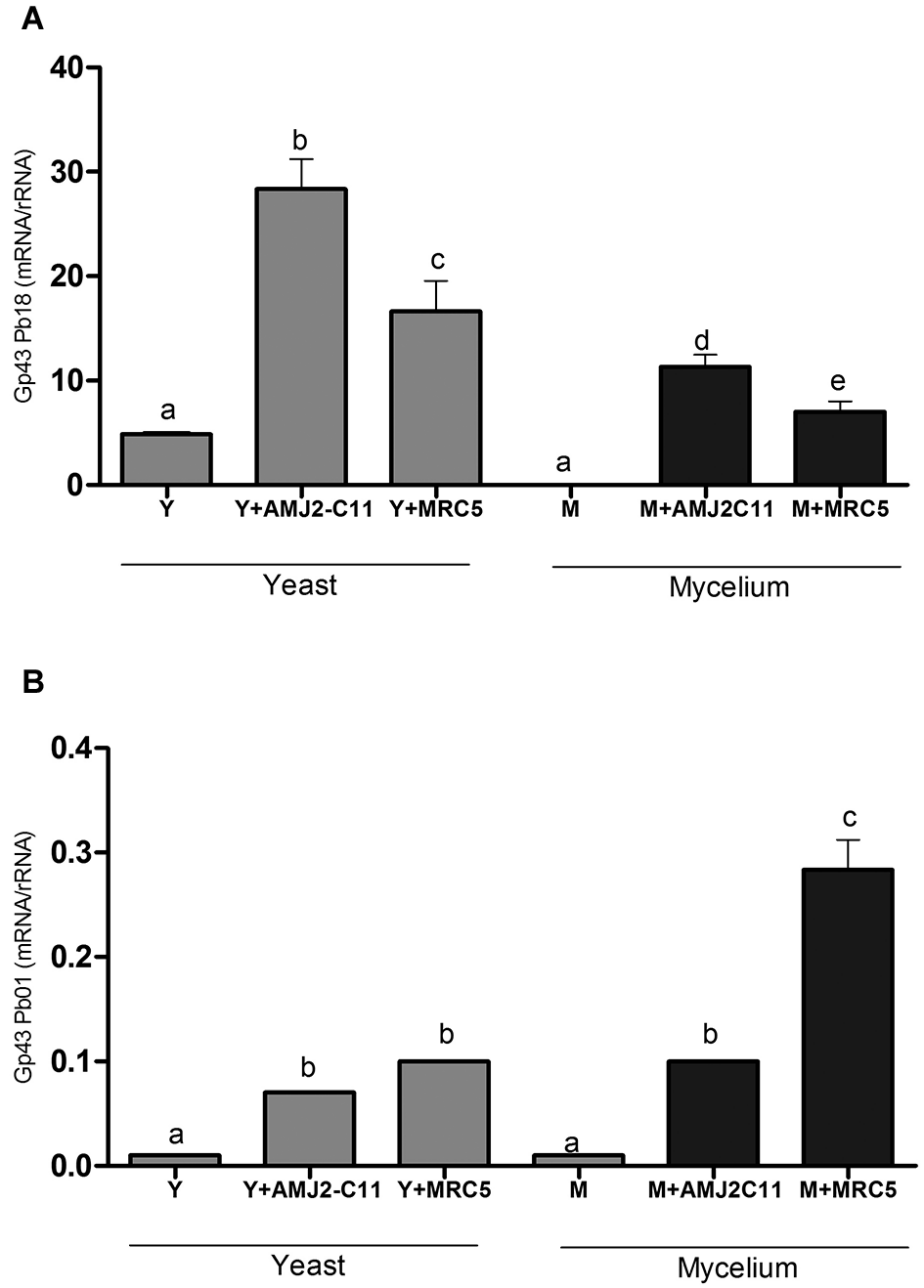
evaluation of gp43 gene expression. Quantitative polymerase chain reaction (qPCR) for (A) *Paracoccidioides brasiliensis* (Pb18) and (B) *P. lutzii* (Pb01). Y form (Y), infected macrophages (Y + AMJ2-C11), infected lung fibroblasts (Y + MRC-5); M form (M), infected macrophages (M + AMJ2-C11), infected lung fibroblasts (M + MRC-5). Analysis of variance (ANOVA) with Tukey’s multiple comparison tests and significance at p < 0.05. *: different letters denote statistical significant.

*qPCR assay* - The expression of the gp43 gene in the Pb18 Y form was significantly higher after macrophage infection than that after lung fibroblast infection (p < 0.05). The results were similar when the M form of the fungus was used, although the expression was lower than that in the Y form (Fig. 1). In contrast, the Pb01 M form showed higher expression of the gp43 gene after lung fibroblast infection than after macrophage infection, although with a ten-fold reduction.

**Fig. 2:**
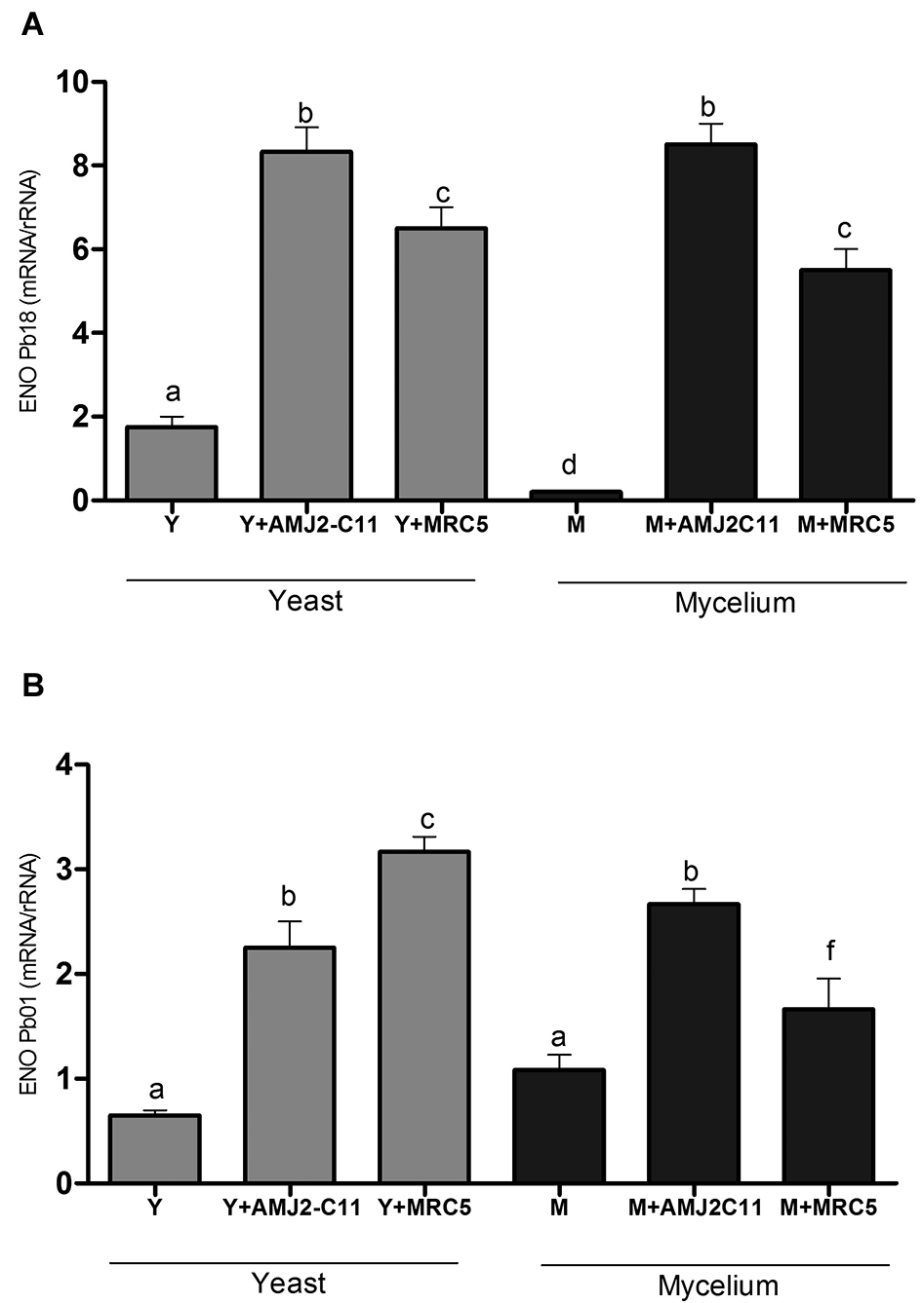
evaluation of ENO gene expression. Quantitative polymerase chain reaction (qPCR) for (A) *Paracoccidioides brasiliensis* (Pb18) and (B) *P. lutzii* (Pb01) Y form (Y), infected macrophages (Y + AMJ2-C11), infected lung fibroblasts (Y + MRC-5); M form (M), infected macrophages (M + AMJ2-C11), infected lung fibroblasts (M + MRC-5). Analysis of variance (ANOVA) with Tukey’s multiple comparison tests and significance at p < 0.05. *: different letters denote statistical significant.

Although there was no difference in ENO gene expression between the Pb18 M and Y forms after infection of either macrophages or fibroblasts, the expression was higher after macrophage infection than after fibroblast infection (Fig. 2). ENO gene expression was lower in Pb01 than in Pb18. In Pb01, the highest expression was found after fibroblast infection with the Y form (p < 0.05).

**Fig. 3:**
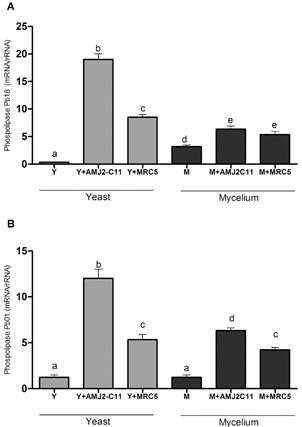
evaluation of phospholipase gene expression. Quantitative polymerase chain reaction (qPCR) for (A) *Paracoccidioides brasiliensis* (Pb18) and (B) *P. lutzii* (Pb01). Y form (Y), infected macrophages (Y + AMJ2-C11), infected lung fibroblasts (Y + MRC-5); M form (M), infected macrophages (M + AMJ2-C11), infected lung fibroblasts (M + MRC-5). Analysis of variance (ANOVA) with Tukey’s multiple comparison tests and significance at p < 0.05. *: different letters denote statistical significant.

Phospholipase gene expression after macrophage infection was considerably higher in the Y form than that in the M form for both species. Phospholipase gene expression in Pb01 was similar to that in Pb18 when comparing the M forms, and lower when comparing the Y forms (Fig. 3). The highest expression was found in the Y forms of both species after macrophage infection. In contrast, the lowest expression was found in the M forms of both species after fibroblast infection.

**Fig. 4:**
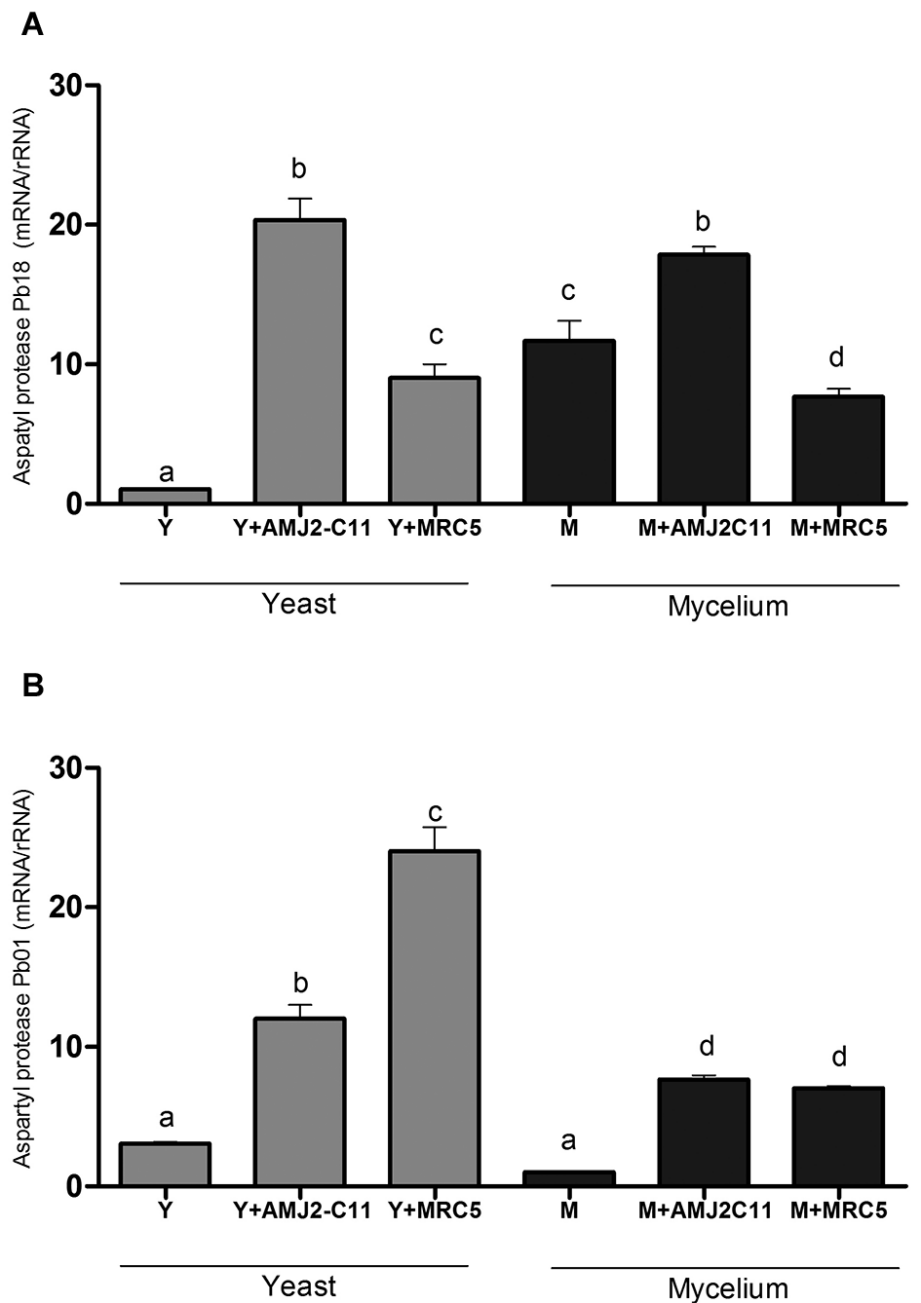
evaluation of aspartyl protease gene expression. Quantitative polymerase chain reaction (qPCR) for (A) *Paracoccidioides brasiliensis* (Pb18) and (B) *P. lutzii* (Pb01). Y form (Y), infected macrophages (Y + AMJ2-C11), infected lung fibroblasts (Y + MRC-5); M form (M), infected macrophages (M + AMJ2-C11), infected lung fibroblasts (M + MRC-5). Analysis of variance (ANOVA) with Tukey’s multiple comparison tests and significance at p < 0.05. *: different letters denote statistical significant.

The Pb18 M form showed significantly higher expression of the aspartyl protease gene than the Y form (p < 0.05). The highest expression of this enzyme was found after macrophage infection with the Y forms (p < 0.05). We found the same level of aspartyl protease gene expression in both forms of Pb01. Fibroblasts infected with Pb01 in its Y form showed the highest expression of this enzyme, whereas, in macrophages, the levels were reduced (Fig. 4).

**Fig. 5:**
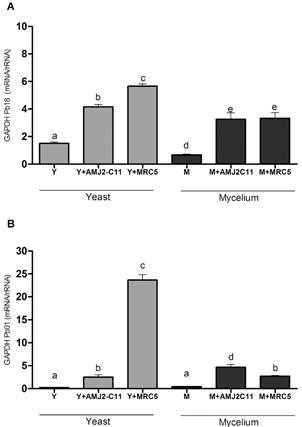
evaluation of GAPDH gene expression. Quantitative polymerase chain reaction (qPCR) for (A) *Paracoccidioides brasiliensis* (Pb18) and (B) *P. lutzii* (Pb01). Y form (Y), infected macrophages (Y + AMJ2-C11), infected lung fibroblasts (Y + MRC-5); M form (M), infected macrophages (M + AMJ2-C11), infected lung fibroblasts (M + MRC-5). Analysis of variance (ANOVA) with Tukey’s multiple comparison tests and significance at p < 0.05. *: different letters denote statistical significant.

GAPDH presented the lowest expression in Pb 18 and Pb 01 in the M and Y forms, whereas this adhesin level was lower in Pb18 infected with both cells. GAPDH gene expression was higher in fibroblasts infected with the Pb01 Y form. Comparatively, the M form showed very reduced levels of GAPDH gene expression (Fig. 5).

**Fig. 6:**
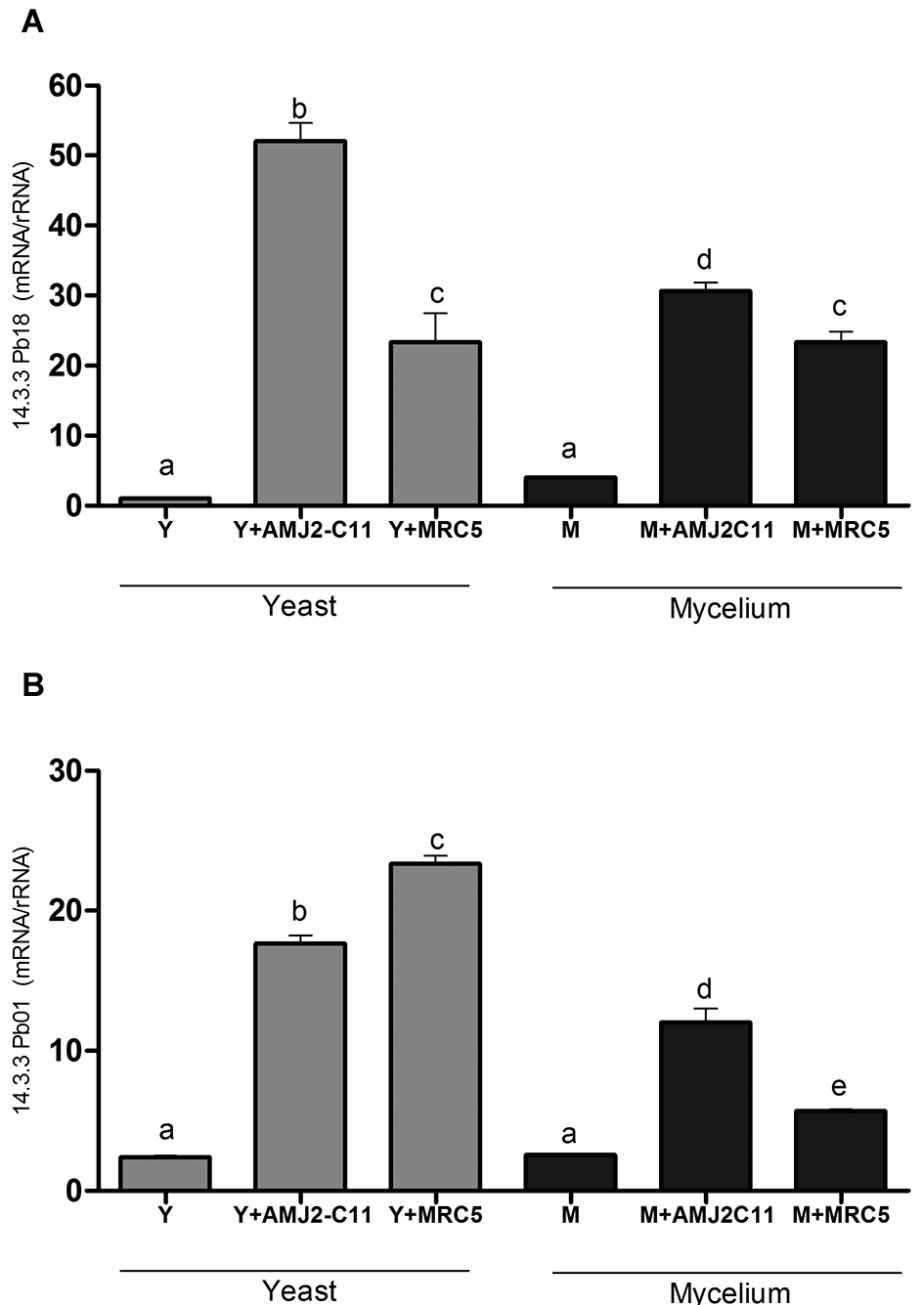
evaluation of 14-3-3 gene expression. Quantitative polymerase chain reaction (qPCR) for (A) *Paracoccidioides brasiliensis* (Pb18) and (B) *P. lutzii* (Pb01). Y form (Y), infected macrophages (Y + AMJ2-C11), infected lung fibroblasts (Y + MRC-5); M form (M), infected macrophages (M + AMJ2-C11), infected lung fibroblasts (M + MRC-5). Analysis of variance (ANOVA) with Tukey’s multiple comparison tests and significance at p < 0.05. *: different letters denote statistical significant.

The highest expression of the 14-3-3 gene was found after macrophage infection with the Pb18 Y form, and the expression was 50% lower in the M form (Fig. 6). This gene had the highest relative level compared to the other genes evaluated. For Pb01, the highest 14-3-3 expression was found after fibroblast infection with the Y form.

## DISCUSSION

The *Paracoccidioides* genus is composed of thermally dimorphic fungi, and the transition from the M to the Y form is essential for disease in humans. Nevertheless, the molecular mechanisms that control this transition still deserve attention, primarily during fungus-host interaction. In the present study, we observed different expression of genes that code for antigens, adhesins, or other potential virulence factors, after macrophage and fibroblast infection with the two forms of two *Paracoccidioides* species.

These experiments can help us understand the mechanisms involved in PCM and the virulence factors of these species. Virulence factors are essential for establishing an invasive fungal infection, including dimorphism, host cell adhesion, cell wall composition, and hydrolytic enzyme production.^16^ ^,^ ^17^ ^,^ ^18^ ^,^ ^19^ ^,^ ^20^ ^,^ ^21^ ^,^ ^40^ Another aspect of this disease is pulmonary fibrosis, which is characterised by excessive deposition of collagen and extracellular matrix components that results in pathological remodeling of the pulmonary architecture.^40^

In this study, the expression of six genes in the Y and M forms of Pb18 and Pb01 after infection of two different cell types was determined using qPCR. First, the highest expression found in the Pb18 M form corresponded to that of the aspartyl protease gene. In contrast, Pb01 expressed relatively few genes and lower levels compared with Pb18.

Among the genes expressed in the Pb18 Y form after macrophage infection, the 14-3-3 gene showed the highest expression, followed by the phospholipase and gp43 genes, and their expression was 50-fold, 10-fold, and 6-fold higher, respectively, than that in the M form. After fibroblast infection with the Y form, the 14-3-3 gene showed the highest expression, followed by the phospholipase and aspartyl protease genes, and their expression was 25-fold, 10-fold, and 10-fold higher, respectively, than that in the M form. The contact of macrophages and fibroblast cells with the Pb18 M form resulted in a similar pattern, with the 14-3-3 gene showing the highest expression, followed by the gp43 gene. ENO and aspartyl protease genes were also expressed after infection of both cell types, and the latter showed higher expression after fibroblast infection. The 14-3-3 gene was not expressed in the M and Y forms of both species.

After infection of macrophages with the Pb01 Y form, 14-3-3 showed the highest expression, followed by the phospholipase and aspartyl genes, and their expression was 18-fold, 12.5-fold, and 6-fold higher than that in the M form. The GADPH, 14-3-3, and aspartyl protease genes showed higher expression levels after fibroblast infection. In general, the 14-3-3 gene showed the highest expression under all conditions, except after fibroblast infection with the M form, where the aspartyl protease gene showed higher expression.

Based on these results, *Paracoccidioides* adhesins are essential for the establishment of infection. They may interact with host cells and establish the colonisation and dissemination of fungi in the host organism,^17^ ^,^ ^41^ interacting with components of the host extracellular matrix (ECM) and lung cells.^41^ Most adhesins are enzymes of the glycolytic pathway, tricarboxylic acid (TCA), and glyoxylate cycle of *Paracoccidioides* spp. They have adhesive properties that facilitate the interaction with the host ECM and act as ‘moonlighting’ proteins.^35^

The 14-3-3 proteins are expressed in most eukaryotic organisms and play varied and crucial roles in a wide range of regulatory processes. These proteins regulate diverse signaling pathways involved in cell survival, cell cycle, and differentiation, affecting their functions via interactions with phosphorylated serine/threonine residues. In *P. brasiliensis*, 14-3-3 was first identified as an overexpressed 30 kDa protein after challenging mice with *P. brasiliensis*.^42^ ^,^ ^43^ Furthermore, Pb 14-3-3 plays an essential role in the adhesion process and is a critical early factor in the development and subsequent progression of invasive infection. Recently, de Oliveira et al.^22^ carried out an in-depth study to elucidate the importance of different adhesins in the virulence of *Paracoccidioides*, and 14-3-3 along with ENO were the most highly expressed adhesins during host-pathogen interactions, which partially supports the results presented in this work. Marcos et al.^35^ demonstrated that downregulation of Pb 14-3-3 expression impairs cell morphology and essential features related to dimorphism, decreases the interaction with pneumocytes, and attenuates the virulence of *Paracoccidioides* spp. in the *Galleria mellonella* model.

As described earlier by our group, this gene is expressed during PCM infection.^43^ Our data in this work also showed high expression of 14-3-3, especially in macrophages infected with the Pb18 Y form. Therefore, this adhesin could be extremely important as a marker of infection. In general, 14-3-3 proteins seem to promote cell survival by binding and sequestering proteins that otherwise activate signaling pathways involved in apoptosis initiation. Indeed, many studies have demonstrated the effects of 14-3-3 proteins on cell death and apoptosis.^44^ ^,^ ^45^

Gp43 acts as an adhesin, favoring the adhesion of *P. brasiliensis* to host epithelial cells.^16^ Studies have reported that GP43 is an immunodominant antigen in *P. brasiliensis*. A modified peptide derived from this molecule has been designed as a vaccine to prevent disease.^46^ ^,^ ^47^ In our study, the gp43 gene showed the highest expression after macrophage infection with the Pb18 Y form. Considering that macrophages are fundamental in controlling fungal infections, the gp43 gene may be involved in a fungal evasion mechanism. According to Popi et al.,^48^ GP43 impairs the phagocytosis of *P. brasiliensis* and inhibits the release of reactive oxygen and nitrogen intermediates involved in the microbicidal activity of macrophages.

Our results also showed significantly lower expression levels of the gp43 gene in Pb01 alone or in contact with both cell types. Studies have demonstrated variation in the gp43 production rate of isolates of different *Paracoccidioides* species, and generally, *P. lutzii* showed low expression.^43^ ^,^ ^49^ There are possible differences in the promoter region of this gene among the different isolates. In addition, there may be interference in post-transcriptional processes, such as RNA interference, which may influence this variability.^50^

Another important and well-described enzyme in *Paracoccidioides* spp. is GAPDH, a key molecule of the glycolytic pathway playing a crucial role in catabolism and carbohydrate anabolism.^51^ In this study, the GAPDH gene showed higher expression in macrophages infected with the Pb01 Y form. Studies performed by Sardi and collaborators.^19^ demonstrated a significant increase in the expression of this gene in *P. brasiliensis* biofilm. Barbosa et al.^51^ found that the GAPDH of *P. brasiliensis* is also located in the cell wall. Thus, it is probably a common receptor of proteins of the extracellular matrix, playing a crucial role in the adhesion of *P. brasiliensis* to host tissues. During copper (Cu) starvation of *P. lutzii* cultures, the expression of GADPH remains at basal levels. However, contact with the host signals the fungus to increase the expression of this protein for adherence to the host, and to obtain the necessary Cu from the host for maintenance. With these results, we can understand how the fungus uses its protein arsenal to adapt to the host and succeed during the infection process.^52^

In our study, the expression of the phospholipase gene was greater after macrophage infection with the Y forms than with the M forms for both species. Phospholipase has been proposed as one of the strategies used by pathogenic bacteria, parasites, and fungi to invade host tissues and establish infection,^18^ ^,^ ^19^ ^,^ ^53^ which would explain the low expression of phospholipase in situations where the fungus is not in contact with cells. Phospholipases are ubiquitous enzymes involved in a wide range of biological functions, such as membrane homeostasis, nutrient acquisition, and the generation of bioactive molecules. These enzymes are known to contribute to bacterial and fungal virulence through various interactions with eukaryotic host cells.^19^ ^,^ ^53^

Another virulence factor addressed in this study was aspartyl protease because proteases from several families are associated with the virulence of human pathogens and play an essential role in the host invasion process of many human fungal pathogens. In our study, this enzyme was expressed in the Pb18 M form. The enzyme increase may be related to fungus pathogenesis. Parente et al.^25^ studied a serine protease and demonstrated that the expression of coding transcripts is induced during the internalisation of *P. brasiliensis* by macrophages.

In conclusion, this study shows that the expression of the genes analysed may increase upon fungal-cell interaction. Therefore, they may be involved in the pathogenesis of paracoccidioidomycosis.
